# On Some Aspects of Memory

**Published:** 1901-02-23

**Authors:** John Aikman


### ON SOME ASPECTS OF MEMORY.

By JOHN AIKMAN, M.D.

III.—ARCHITECTURE—(*Continued*).

THERE are branches which put forth no leaves because of the incapacity, indolence, or inattention of the owner. There are branches from which the leaves disappear from disuse. There are branches which are leafless and often distorted—the result of bodily disease, or the abuse of alcohol, or narcotic drugs. And lastly, there is the withered branch of old age. But through all these runs the idea of poverty, unavoidable or reprehensible. Evidence that in some degree "Man is master of his fate." And there is this difference between these upper branches and the root fibre, that the branches and leaves make the memory record, the root fibre only transmits it. That the root fibre is hardly affected by the force of the impression, the brain cells, branches, and leaves, are. And that our faculty of attention rules the record made. But the root fibre, the cell body, the branches and buds, together form the machine which we call a "neuron."

When we examine the hard root process, usually called the "Axon" we find that it is the beginning of a nerve fibre, which connects the brain cell with the various parts of the body. Its sheath is a living one which grows with use, and has the power of repairing injury. (The sheath of Schwann.) This sheath contains a fluid, a compound of fat and phosphorous, not very unlike the yolk of an egg. The sheath seems to have the power of increasing the sensitiveness of the nerve fibre. For example, when we have learnt to hear a sound, which at first was so slight that it passed unnoticed, or to see a shade in colour, or recognise the quality of a fabric by touch, we know that the nerve sheath has become stronger; otherwise expressed, the stronger the sheath, the more completely is the nerve conductor insulated. We improve our faculty in those directions by the attention which we give to our newly acquired knowledge, and the effect which it produces upon the upward processes of our nerve cells.

There is much that is interesting in the study of the forces which travel along nerves, towards the brain.

The simplest and one of the earliest ideas of a memory made is furnished by the sense of touch. The extremity of the nerve of touch at the tip of the finger is buried in a lemon-shaped body. When we press with the finger the touch bulb squeezes the nerve ending and the fact is transmitted to, and registered in, the brain.

Taste is but a modification of the same act, by a special arrangement of the nerve endings. We know that the nerve of bitter taste is placed far back in the tongue, and that the recognition of sweetness is nearer the tip. Many different substances taste sweet; we cannot tell why. They have only one point of agreement, that in their chemical composition the atoms of carbon run in multiples of three. If our tongues can appreciate that circumstance they have more discrimination than is usually accorded to them. Still it is true that cane sugar contains 12 atoms of carbon, grape sugar 6 atoms, milk sugar 12 atoms, malt sugar 12 atoms, saccharine 6 atoms, and glycerine 3 atoms. (*Lancet*, May 26, 1900, p. 153).

Smell weighs ponderable particles and records the result. In these three senses there is obvious contact. But all our senses are avenues of knowledge to the brain —at once the feeders and the excitors of memory.

Hearing and sight do not obviously imply contact. We may reach the consideration of them more readily by some remarks upon the perception of heat.

"It is a good sign to have one's feet grow cold when he is writing. A great writer once told me that he often wrote with his feet in hot water; but for this, all his blood would have run into his head, as the mercury sometimes withdraws into the bulb of the thermometer." ("Autocrat of the Breakfast Table," p. 13). The illustration is a happy one. We know that mercury expands with heat and contracts with cold. Physicists tell us that heat is motion, and the measure of heat is the measure of motion. That in heated bodies the particles revolve more rapidly and repel each other. If a nerve fibre is heated, its fluid contents expand, and the expansion is registered *in* the brain cell, and *upon* our consciousness. Cold is a negative quantity, that is to say an absence of heat, lessened movement, lessened repulsion of particles, and consequent retraction. We shrink with cold, but we remember it less than we do the sensation of heat. We have no doubt that the laws of physics underlie the actions of living organisms, but pressure and weight, heat and cold do not explain all the phenomena. To what we do not know we give the term Chemio (or Chemo) taxis. The word is a name for the unexplained. Its first half appears in the classic languages as a derivative from the Aryan, and means "a hidden art." Hieroglyphic readings indicate the land of Chem as the "land of mystery." The second half is more modern, and in the Greek tongue means to set in order of battle, or to array for action. It lives in the military speech of the present day in the word "tactics."

"Chemotaxis" means "by an unknown power I set in order for action."

Have we any glimpses of how sound and light arrange living particles, or dead matter?

Most people know something about sound. They know that it consists of vibrations or waves. They know that in musical sounds the waves follow one another at regular intervals. That a low bass note has about 80 vibrations per second, and a high treble note about 1,000. That the concert pitch of the treble C is 512 vibrations per second, and that each octave has just twice as many or half as many vibrations as the octave below or above it. Some of the better informed may know the exact arithmetical ratio in point of vibrations of the various notes in the major and minor scale, and yet be unaware of how sound arranges particles of matter.

If you scatter some sand on a metal plate, and draw the bow of a violoncello across the edge of the plate, you produce a musical note, and the sand arranges itself in lines. If you press your finger on the edge of the nodal line you produce the octave, and get a new set of lines. If you divide the plate unequally you get new lines of sand, and so on for all divisions and combinations of the notes of the octave. Obviously then the number of vibrations per second is not the whole measure of a musical note.

But that is not all. If in place of sand which is heavy, you use a lighter powder, such as Lycopodium, the lines assumed by the Lycopodium are quite different, note for note, to those assumed by the sand. A creamy substance gives a still different set of lines. That wood fibre is capable of some such arrangement is shown by the history of violins. A new violin of the best make is a poor instrument even in skilled hands. Its maker subjects it to hours and weeks of strumming before he offers it for sale. The result is that the particles of its wood fibre arrange themselves as the powders do upon the metal plate. It is then capable of giving forth melody which an audience attributes to the performer, but which is in great part but the roused memory of less perfect handling.

(*To be continued.*)

